# Strengths and Limitations of Web-Based Cessation Support for Individuals Who Smoke, Dual Use, or Vape: Qualitative Interview Study

**DOI:** 10.2196/43096

**Published:** 2023-12-08

**Authors:** Laura Struik, Kyla Christianson, Shaheer Khan, Ramona H Sharma

**Affiliations:** 1 School of Nursing University of British Columbia Okanagan Kelowna, BC Canada; 2 School of Health and Exercise Science University of British Columbia Okanagan Kelowna, BC Canada; 3 School of Social Work University of British Columbia Okanagan Kelowna, BC Canada

**Keywords:** qualitative research, tobacco use, smoking, vaping, cessation, eHealth, individuals who smoke, users, tobacco, e-cigarettes, cigarettes, web-based, support, behavioral, smartphone app, social media, mobile phone

## Abstract

**Background:**

Tobacco use has shifted in recent years, especially with the introduction of e-cigarettes. Despite the current variable and intersecting tobacco product use among tobacco users, most want to quit, which necessitates cessation programs to adapt to these variable trends (vs focusing on combustible cigarettes alone). The use of web-based modalities for cessation support has become quite popular in recent years and has been compounded by the COVID-19 pandemic. Therefore, understanding the current strengths and limitations of existing programs to meet the needs of current various tobacco users is critical for ensuring the saliency of such programs moving forward.

**Objective:**

The purpose of this study was to understand the strengths and limitations of web-based cessation support offered through QuitNow to better understand the needs of a variety of end users who smoke, dual use, or vape.

**Methods:**

Semistructured interviews were conducted with 36 nicotine product users in British Columbia. Using conventional content analysis methods, we inductively derived descriptive categories and themes related to the strengths and limitations of QuitNow for those who smoke, dual use, or vape. We analyzed the data with the support of NVivo (version 12; QSR International) and Excel (Microsoft Corporation).

**Results:**

Participants described several strengths and limitations of QuitNow and provided suggestions for improvement, which fell under 2 broad categories: *look and feel* and *content and features*. Shared strengths included the breadth of information and the credible nature of the website. Individuals who smoke were particularly keen about the site having a nonjudgmental feeling. Moreover, compared with individuals who smoke, individuals who dual use and individuals who vape were particularly keen about access to professional quit support (eg, quit coach). Shared limitations included the presence of too much text and the need to create an account. Individuals who dual use and individuals who vape thought that the content was geared toward older adults and indicated that there was a lack of information about vaping and personalized content. Regarding suggestions for improvement, participants stated that the site needed more interaction, intuitive organization, improved interface esthetics, a complementary smartphone app, forum discussion tags, more information for different tobacco user profiles, and user testimonials. Individuals who vape were particularly interested in website user reviews. In addition, individuals who vape were more interested in an intrinsic approach to quitting (eg, mindfulness) compared with extrinsic approaches (eg, material incentives), the latter of which was endorsed by more individuals who dual use and individuals who smoke.

**Conclusions:**

The findings of this study provide directions for enhancing the saliency of web-based cessation programs for a variety of tobacco use behaviors that hallmark current tobacco use.

## Introduction

### Background

Tobacco use remains as one of the most serious public health issues worldwide. People who use tobacco products are more at risk for developing severe health conditions, including chronic obstructive pulmonary disease, heart disease, cancer, and diabetes [1]. It has been estimated that tobacco accounts for approximately 48,000 deaths each year in Canada [2]. In 2020, 12% of Canadians aged ≥15 years reported using at least 1 type of tobacco product in the past 30 days [3]. Tobacco use behavior has shifted in recent years, particularly since the introduction of e-cigarettes; smoking rates have decreased, and e-cigarette use (also known as vaping) rates have increased. According to the 2020 Canadian Tobacco and Nicotine Survey, smoking rates for those aged ≥15 years were at 10% (decreased from 12% in 2019), but vaping rates were at 17% (increased from 16% in 2019) [3,4].

Population-based surveys, such as the Canadian Tobacco and Nicotine Survey, reveal the complexity of the tobacco use landscape through differential use behaviors across age demographics. Youth are more likely to exclusively use e-cigarettes, older adults are more likely to exclusively smoke, and young adults have the highest rates of dual use (using both combustible cigarettes and e-cigarettes) [3,5]. Although vaping has potential as a form of harm reduction for adult individuals who smoke, it has become most popular among youth, particularly nonsmoking youth, in large part owed to the appealing marketing tactics used by the e-cigarette companies to entice youth to use their products [4,6]. Among individuals who vape in Canada who have never previously smoked, most (74%) were aged between 15 and 19 years [4]. The uptake of vaping among youth is particularly concerning, given that vaping has been associated with an increased risk for lung and cardiovascular disease, cancer, and nicotine addiction (especially from popular high dose nicotine devices), and may encourage youth to transition toward smoking combustible cigarettes [7-11]. According to a recent meta-analysis, the use of e-cigarettes at a young age was found to increase one’s risk for cigarette smoking later in life by 2.3 to 12.3 times [12].

In addition, there is evidence indicating that sex and gender influences tobacco use. For example, previous explorations of smoking behaviors and intentions to quit have found differences among adult men and women, with men tending to demonstrate high rates of smoking behaviors, such as daily use of nicotine products, and women being less likely to successfully quit despite equivalent desires, intentions, and attempts to quit when compared with men [13,14]. Women also tend to face great internal barriers to cessation, such as stress and emotional setbacks, tend to respond more to environmental cues and stress-related triggers associated with nicotine addiction, and may have differential pharmacological responses to nicotine addiction, such as susceptibility to specific nicotine-related side effects, including depression, weight loss, and higher levels of negative affect [15,16]. Although limited, there are some studies indicating that gender-varying people demonstrate an increased risk for nicotine use and associated health consequences [17]. In this regard, it is important to pay attention to gender influences when exploring the cessation needs of various nicotine product users.

Tobacco use behaviors have also been influenced by the recent COVID-19 pandemic. Researchers have found inconsistent results regarding tobacco use behaviors (eg, some have increased, some have decreased, and some have not changed), which is owed to variability in reporting time, tobacco product type, and demographic characteristics [18]. However, most users consistently have the desire to quit. A 2019 report from the University of Waterloo (Tobacco Use in Canada: Patterns and Trends) highlights that 57.9% of individuals who smoke were considering quitting in the next 6 months [19]. Moreover, in 2020, 39% of individuals who smoke and 37% of individuals who vape aged ≥15 years in Canada made a quit attempt lasting 24 hours [4]. Despite the willingness to quit, in the absence of cessation support programs, the World Health Organization concludes that only 4% of attempts to quit tobacco will succeed, highlighting the need for cessation support [20]. The World Health Organization goes on to say that professional support and cessation programs can double users’ likelihood of quitting [20]. The ever-evolving tobacco use landscape necessitates the availability of tobacco cessation programs that accommodates the variable use patterns that hallmark tobacco use currently.

Propelled by the COVID-19 pandemic, more health care services are being accessed remotely, including nicotine cessation supports. Although in-person cessation services remain available, engagement rates have declined since the pandemic. A 2021 study examined engagement rates in Smoking Treatment for Ontario Patients, an Ontario-based, in-person smoking cessation service [21]. The authors concluded that, owing to the COVID-19 pandemic, enrollment decreased; in March 2020, enrollments were 69% lower and visits were 42% lower than the average for the 2 previous years [21]. The declining in-person engagement rates owing to COVID-19 expose the need to have robust and adaptable methods for tobacco users to gain access to support, particularly web-based modalities of tobacco cessation support, which align with the needs and preferences of current tobacco users [22,23]. Web-based modalities have several benefits for delivering smoking cessation support, including enhanced reach, accessibility, and anonymity [24]. In this regard, leveraging web-based and remote modalities for tobacco use cessation services is a timely priority.

The government of Canada funds and supports the delivery of web-based and telephone-based smoking cessation programs. In British Columbia, QuitNow is the largest digitally mediated smoking cessation service. Given that the government aims to reduce tobacco use prevalence to 5% by the year 2035, this necessitates studies of the effectiveness of smoking cessation programs that use digital technologies [7]. Given the shifting tobacco use landscape and rise in e-cigarette and dual use, there is a need for research on how cessation programming is meeting the needs of these diverse end users. Understanding this will help pave a way forward to meet the needs of current tobacco users. The purpose of this qualitative descriptive study therefore was to understand the strengths and limitations of cessation support offered through QuitNow from the perspectives of a variety of end users who smoke, dual use, or vape.

### About QuitNow

QuitNow [25] is a free-of-charge, 24/7, web-based resource that provides support for quitting or reducing tobacco and e-cigarette use. The program is funded by the province of British Columbia and managed by the BC Lung Foundation. QuitNow helps British Columbians quit tobacco by providing user-customized supports upon sign-up. Supports include personalized information and quit plan options; free sessions with a trained quit coach; a coach-moderated community forum; telephone, SMS text message, and live-chat support; information for loved ones; resources for health care professionals; region-specific help directories; and general information about nicotine product use and cessation.

## Methods

### Recruitment

Residents of British Columbia who were motivated to quit vaping or smoking were recruited through the use of web-based recruitment advertisements on Facebook and Instagram, posted by a third party (PH1 Research) that could target population groups. Eligibility criteria included individuals who smoked or vaped, were interested in quitting, and could communicate in English. Individuals were excluded from the study if they were aged <16 years, were not using e-cigarettes or traditional cigarettes, and were in a special patient population (eg, patients with cancer and pregnant women).

### Data Collection

All consenting participants completed a brief demographic survey, familiarized themselves with the QuitNow website if they were not current users, and then participated in an approximately 60-minute semistructured interview via Zoom (Zoom Video Communication) at the University of British Columbia. During these interviews, we asked participants about what they liked and disliked about QuitNow and what suggestions they had for improving QuitNow to better meet their needs.

### Data Analysis

Our data set consisted of 36 participant interviews, which comprised 12 (33%) cigarette-only users (individuals who smoke), 12 (33%) e-cigarette-only users (individuals who vape), and 12 (33%) individuals who dual use, with 50% (6/12) men and 50% (6/12) women per category. This approach was undertaken to provide an equitable representation across the different nicotine user types and genders represented in the large participant pool (no nonbinary or gender-nonconforming individuals were present in the recruitment pool), ensuring a balanced perspective about the represented group’s behaviors and experiences. This targeted approach further allowed for a comprehensive exploration of any potential differences or similarities among individuals who smoke, individuals who vape, and individuals who dual use, thereby improving the depth and robustness of our findings.

Interviews were audio recorded and transcribed by the research team. We used inductive content analysis [26] and NVivo (QSR International) and Excel (Microsoft Corporation) software to organize the large data set. First, we transcribed the audio recordings. The research team completed the transcription, enabling familiarization of the data among the team. Then, we engaged in 3 collaborative coding sessions via Zoom at the University of British Columbia to develop the initial codebook for application to the entire data set. During the first collaborative session, each team member involved in analysis was responsible for reading the same 2 transcripts and discussing the process of assigning codes and subcodes. During the second session, each team member independently coded another set of 2 transcripts. Once the working analytical process and framework were established, the research team applied the framework to 1 transcript from a demographic subset to ensure the applicability and flexibility of the framework. The third session focused on the results of this process, and the team members discussed and agreed on the final analytical framework and codebook for use on the remaining transcripts. With the oversight of the project coordinator (RHS) and lead author (LS), research assistants then applied the codebook to subsets of the total sample (eg, one research assistant coded youth male and female interviews, whereas another coded those of individuals who smoke only). This way, we were able to ensure that variations in contexts, perspectives, and experiences that might influence their responses were brought forward. The data were then charted onto Excel to enable the research team to compare and contrast the findings across the entire sample, across nicotine user types, and across genders.

### Ethical Considerations

All participants provided written consent via email before commencing the interviews and were informed that their participation was voluntary through the consent form and then reiterated before each interview. Each participant received an electronic gift card worth CAD $50 (US $36.45) to thank them for their contribution to the study. The study was approved by the Behavioral Research Ethics Board at the University of British Columbia’s Okanagan campus (H21-00145). Participants were given an identification number to anonymize their responses.

## Results

### Sample Characteristics

This study included 36 individuals, with age ranging from 17 to 58 years. All male participants identified as men and all female participants identified as women. The sample was split evenly among individuals who smoke, individuals who vape, and individuals who dual use (12/36, 33% each) and between men and women (18/36, 50% each). Most participants were not of Indigenous heritage (25/36, 69%) and did not use QuitNow at the time of data collection (32/36, 89%). In addition, most participants (21/36, 58%) considered themselves somewhat addicted to nicotine, followed by 39% (14/36) identifying as very addicted and only 3% (1/36) stating that they were not at all addicted. A complete breakdown of participant demographics is outlined in Table 1.

**Table 1:** Participant demographics (N=36).

Demographic variables		Participants, n (%)
**Age (years)**		
	16-18	6 (17)
	19-24	11 (31)
	25-29	7 (19)
	>30	12 (33)
**Sex (gender)**		
	Male (men)	18 (50)
	Female (women)	18 (50)
**Indigeneity**		
	Indigenous	11 (31)
	Non-Indigenous	25 (69)
**QuitNow user status**		
	User	4 (11)
	Nonuser	32 (89)
**Nicotine user type**		
	Cigarette-only user (individual who smokes)	12 (33)
	Vapor product–only user (individual who vapes)	12 (33)
	Cigarette and vapor product user (individual who dual uses)	12 (33)
**Nicotine addiction severity**		
	Not at all addicted	1 (3)
	Somewhat addicted	21 (58)
	Very addicted	14 (39)
**Cigarette use duration**		
	6 months to 1 year	1 (3)
	1-2 years	4 (11)
	3-5 years	4 (11)
	>5 years	17 (47)
**Vapor product use duration**		
	<6 months	1 (3)
	1-2 years	11 (31)
	3-5 years	8 (22)
	>5 years	4 (11)

### Qualitative Findings

#### Overview

**Figure 1:**
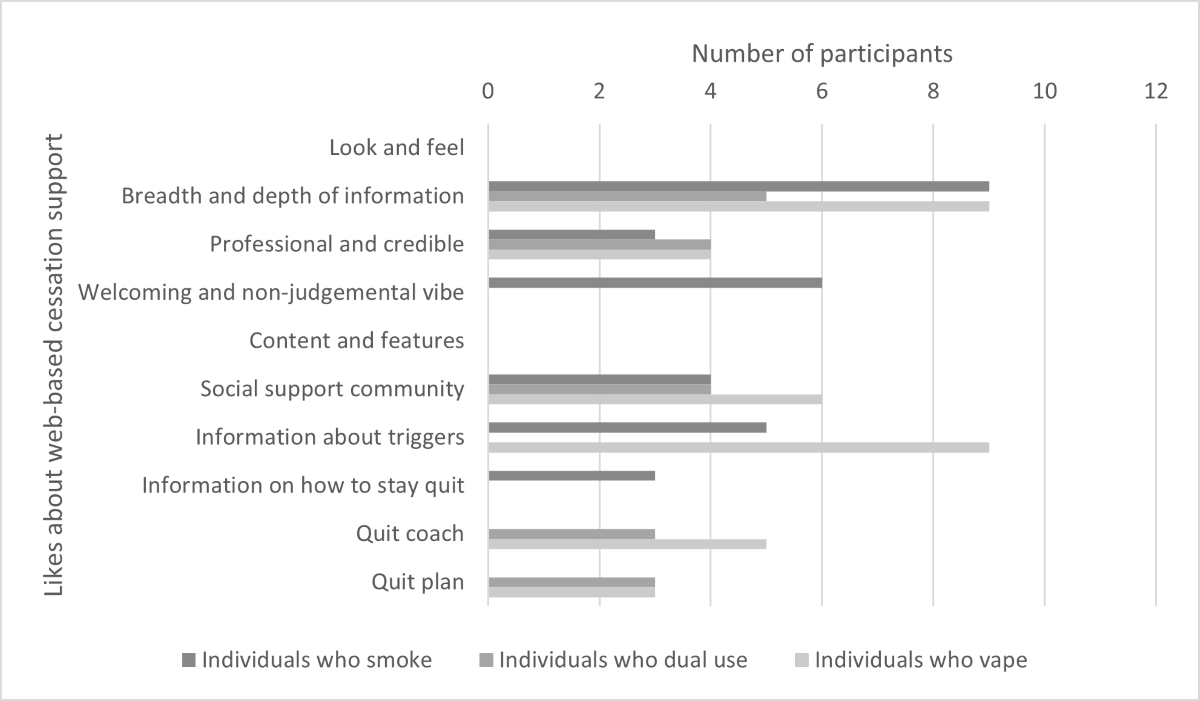
Likes about web-based cessation support (QuitNow).

**Figure 2:**
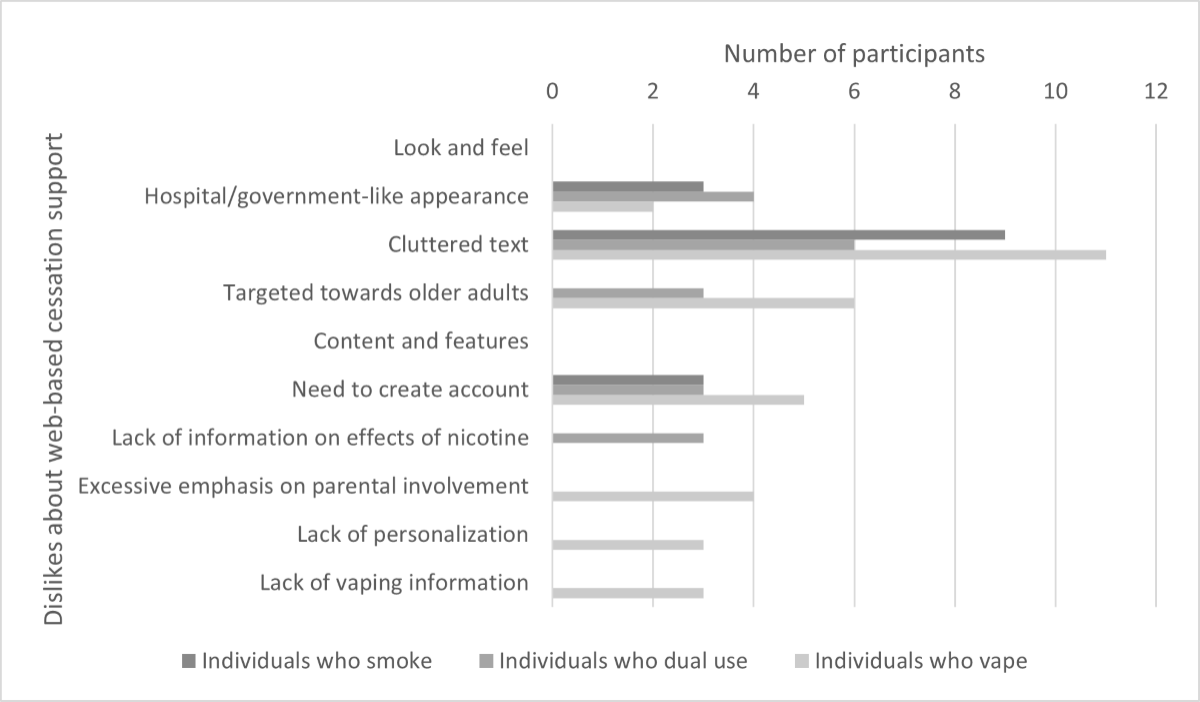
Dislikes about web-based cessation support (QuitNow).

**Figure 3:**
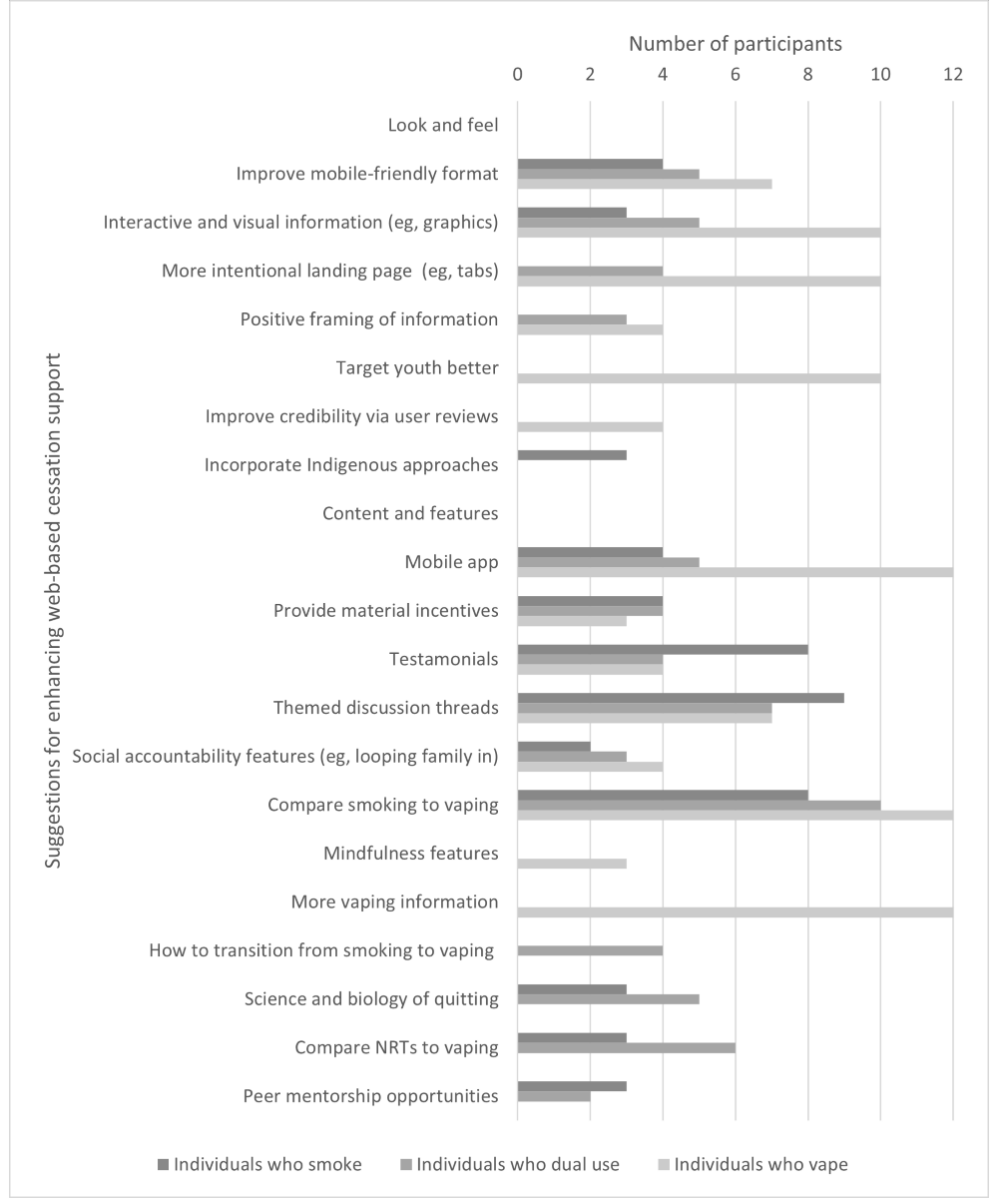
Suggestions for enhancing web-based cessation support (QuitNow).

Participant responses related to the following 3 major categories: likes about QuitNow, dislikes about QuitNow, and suggestions for QuitNow. Data in these categories were further broken down into the following 2 subcategories: look and feel of QuitNow and content and features of QuitNow. These categories and subcategories were examined with respect to nicotine user type (individuals who smoke, individuals who vape, and individuals who dual use) and gender (men and women). Figures 1-3 provide an overview of emergent themes within the abovementioned categories and subcategories with respect to nicotine user type and the whole sample; no significantly unique nuances in endorsed topics were found between males and females.

#### Likes About Web-Based Cessation Programing (QuitNow)

Participants expressed several favorable views regarding the look and feel of QuitNow, which included the broad scope of information available to them, its professional appearance, and the welcoming and nonjudgmental feeling it embodied. Regarding the breadth of information, all participants (36/36, 100%) expressed appreciation for all the various information and support that were freely available to them on the website, which enabled them to pick and choose information that was relevant to them during their quit journey:

First of all, they have, like, why you should quit? And how you should quit? And all the questions that you might have, while in the process of quitting.... And the best thing is that it’s free. So everyone willing to quit, can access the site...

#60; male; individual who vapes

Regarding professional appearance, participants from all user groups appreciated the credibility reflected in the site through government backing, providing evidence-based information, and ensuring that the site did not contain advertisements. All these factors were described as key to trusting that the site was genuinely there to support their quit efforts (vs containing ulterior motives, such as financial gain from advertisements). The welcoming and nonjudgmental look of the website was another notable strength, identified specifically by half of the individuals who smoke (6/12, 50%):

The website itself almost creates a vibe that they’re there for you even though you don’t know who they are.

#9; female; individual who smokes

This atmosphere made them feel accepted and understood, which they said is critical for users dealing with the stigmatization often associated with smoking.

Regarding content and features, a significant strength identified across user groups was the social support community, whereby users can exchange support with each other on a forum:

Definitely the service groups and the community where you can talk to others and also learn how to stay on target after you quit to prevent relapses, that’s what I like about it.

#24; male; individual who smokes

Among specific user types, individuals who smoke were particularly keen on receiving information about how to stay quit and on receiving information about triggers, whereas individuals who vape only spoke about the latter as a strength. Both individuals who dual use and individuals who vape appreciated the quit coach and quit plan, but individuals who smoke did not mention these functions when describing what they liked about QuitNow.

#### Dislikes About Web-Based Cessation Programs (QuitNow)

Participants identified several areas of dissatisfaction regarding the look and feel of the web-based cessation programs (QuitNow). Although the participants appreciated the credibility of the site, many (9/36, 25%) mentioned that they did not like the institutional look and feel of the site, often referred to as hospital-like or government-like appearance. In addition, individuals across user types lamented the cluttered text. They described how the large amount of information (identified as a positive aspect) presented primarily through text was a major limitation because it limited their ability to navigate the site and access the information in a timely manner:

I think the front page is a little bit crowded right now. I think simplicity is important to just get straight to the point and not have to spend too much time scrolling through things.

#57; male; individual who dual uses

Finally, individuals who dual use and individuals who vape described the site as tailored to older adults versus youth and young adults, which limited its appeal to the young demographics.

Regarding content and features, many users (11/36, 31%) across user groups did not like that they needed to create an account to access all the support available on the site. They explained that, in addition to concerns around identity protection, it was not clear why an account was necessary or beneficial to them (eg, what features would they be able to access):

It did press you to make an account.... I didn’t necessarily want to make an account...

#1; male; individual who vapes

In terms of content, the primary concern was related to insufficiency of certain types of information. Specifically, individuals who dual use did not think that there was sufficient information about how nicotine operates on the brain and body, which left them feeling as if they did not understand the mechanisms behind addiction, ultimately preventing them from understanding their quit journey:

I think there could be more information on how nicotine interacts with the brain. Some studies say it’s not the nicotine after a point, it’s the addiction and stimulants and stimulation. I don’t understand any of it, because they don’t have a lot of information on how nicotine reacts with the brain and body. Everybody knows the health risks and stuff like that, but people want to find a reason why [the addiction is] happening, it’s interesting, it’s motivating.

#40; male; individual who dual uses

In contrast, individuals who vape described a lack of information about vaping specifically (vs smoking or general nicotine use more broadly):

You know that cigarettes are really bad for you. Over a century of research and whatever indicates you get black lungs from smoking...lots of people know that. But vaping, I feel like I haven’t seen a lot of research...it’s not as publicly shown or indicated that it’s a “proven” bad. Also, there’s a lot of online reviews for vapes, so people sometimes see it as a positive thing, like on YouTube. Obviously, there’s two sides to it, there are people who got hospitalized for having popcorn lungs from vaping or whatever, but publicly, it’s not broadcasted that vaping is really bad for you. So, I do think that supports need to have a pull to make people actually want to quit vaping...vaping is less stigmatized than smoking, so educating people on the actual harmful effects of vaping and using that information to convince them why it’s worth it to quit - the same as cigarettes.

#63; male; individual who vapes

In addition, some individuals who vape believed that there was excessive emphasis on involving parents in the quitting process, which was perceived as a negative aspect. They explained that involving parents would likely come with more shame than support:

Telling your family is not really a feasible option for a younger demographic that doesn’t want to go to their parents and say, “Hey, I vape. Can you help me stop?”.... I know that in my particular situation if I were to tell my parents that would surround a lot of shame and guilt as opposed to...I wouldn’t be able to seek support, I would just be getting blame instead.

#10; female; individual who vapes

Finally, individuals who vape explained that there was a lack of more personalized information and opportunities to generate more personalized information to support them in quitting. No explicit dislikes regarding content and features were recorded among the individuals who smoke, potentially indicating general satisfaction with the current state of the program for this user group.

#### Suggestions Regarding Look and Feel to Enhance Web-Based Cessation Programs (QuitNow)

Participants had a wide variety of suggestions for improving web-based cessation programs. Regarding the look and feel, participants across user groups agreed that web-based programs had to be more mobile friendly because most individuals were accessing websites on their mobile phones:

An easier way to access QuitNow is by phone, or like using an app and using your laptop, and it’s the same with lots and lots of things. So, I think overall, phone.

#63; male; individual who vapes

In addition, regarding information delivery, participants strongly advised for increased incorporation of visual, engagement-focused tools, such as images and infographics, rather than an overreliance on text. Although shared across user types, this suggestion was particularly prominent across the entire set of individuals who vape:

There’s lots of text, and it’s small too...for most people that’s not going to be thorough reading that on a mobile device. So, try to attend to more engaging features like more images, more diagrams or visual comparisons...just make it more interactive and less text wall.

#46; female; individual who vapes

In addition, individuals who vape and individuals who dual use were vocal about the need for more intuitive organization of the large amount of information available at the outset via the landing page. Participants made several suggestions around generating tabs and a table of contents for organizing this information:

Right now, there’s so much information on the main page, everything is on the same page and it’s a bit overwhelming. So maybe making like three or four major tabs, or a more central landing page with an overview of what all their services are, that would make it a lot less confusing.

#12; male; individual who dual uses

Regarding the credibility of the site, individuals who vape said that this could be enhanced by incorporating user reviews on the site:

I would like to see a little bit of people’s reviews of the website itself, for example how the meetings with the coaches go, how the features run, etc.

#81; female; individual who vapes

Participants made suggestions around how to tailor the content and features within the site and include additional content and features that would benefit their quit journey. Participants suggested incorporating Indigenous approaches to cessation (eg, incorporating cultural practices), making the content more youth friendly (eg, tapping into youth cultures), and ensuring that the content is framed in a positive way (eg, benefits of being nicotine free vs negative aspects of addiction).

#### Suggestions Regarding Content and Features to Enhance Web-Based Cessation Programs (QuitNow)

Participants offered several insightful suggestions to enhance the content and features of the web-based cessation programs (QuitNow; Figure 3). All types of users suggested that the website be complemented with an app, so that they could access QuitNow and track their quit journey more easily:

I think an app. Like if Quit Now had an app that you could track yourself with, like how long it’s been since your last cigarette and stuff like that.

#59; male; individual who smokes

Individuals who dual use and individuals who vape were more likely to make this suggestion, especially individuals who vape.

Regarding information, a common suggestion across all user groups was to better differentiate vaping and smoking while providing equivalent types and amounts of information for both. Individuals who smoke and individuals who dual use specifically suggested displaying vaping-specific information in a separate section, and individuals who dual use suggested providing information about how to transition from smoking to vaping:

When people switch to vaping from smoking...you can vape indoors so sometimes that ends up with more nicotine use happening.... But I guess you can control how much nicotine you put in it, like you can go down to nothing, so that’s good. But more information would be good I think.

#68; female; individual who dual uses

In contrast, individuals who vape unanimously requested more information pertaining to quitting vaping:

Vaping and e-cigarettes is a new topic so there’s not a lot of information. Personally, I have done research on it but haven’t seen that there’s been some negative research on it. Obviously, it’s still very new, so I understand why it’s there’s not a lot of writing and information. But I guess as time goes on, the more information the better. Just more information in general and on the harmful effects, long-term effects, short-term effects.... That would be helpful.

#31; male; individual who vapes

The inclusion of more scientific information and resources was also recommended by individuals who dual use and individuals who smoke, emphasizing the desire for evidence-based resources in their quitting journey:

I would like to see the scientific like aspect of quitting. Like the harm that smoking does, and learning about the recovery process and how our body goes through that.

#27; male; individual who dual uses

Regarding additional features, the introduction of more goal-based and incentive-based features was a prominent suggestion. This was most highly recommended by individuals who dual use, who suggested implementing progress trackers, providing material incentives, and incorporating workout subscriptions into the website membership. Although individuals who vape endorsed this extrinsic (external motivation) approach to quitting, individuals who vape were the only group to also suggest a more intrinsic (internal regulation) approach to quitting through the use of mindfulness and journaling features:

What if there was a section on [a QuitNow app] where you could type what was making you want to smoke or vape, like writing in a journal about a stress-inducing situation that you wanted to vent about...plus stuff like mindfulness or meditation activities to distract you from smoking or vaping.

#10; female; individual who vapes

Participants suggested various ways to enhance the social elements of the website. All types of users suggested the incorporation of testimonials from others into the site:

I’d want to see testimonials of user experiences and what helped or how [QuitNow] may not have helped.

#81; female; individual who vapes

All types of users also suggested incorporating more social accountability features into the site, such as enabling family members to access a user account or join in telephone or Zoom calls with quit coaches. Individuals who smoke and individuals who dual use suggested incorporating more peer-to-peer mentorship opportunities, such as through in-person and web-based support groups:

I don’t know that much about AA or drug rehab, but when you get a group of people together with the same interests, they really benefit each other. So, some sort of user group meetup, whatever it is, would be awesome. If you’re hanging out with smokers, or drinkers or whatever, it’s more likely, in my case at least, that I’m going to have a cigarette or I’m going to have a drink. If I’m hanging out with people that understand how hard it is to quit, that probably quit many times before - as every smoker has - I would think that would be very, very beneficial.

#39; male; individual who smokes

Finally, all types of users suggested creating themed discussion threads on the community forum, so that they could access the social support that was most relevant to them (eg, based on nicotine product type, gender, age, and quit status):

I think [themed discussion threads] are so important because you need that support especially after hours when there’s nobody around that’s available...but and that’s when you smoke the most, at night or when something happens in your life... .And the different themes could be different options for different people so maybe a women’s thread, one for men, one for trans people...

#75; female; individual who smokes

## Discussion

### Principal Findings

This study serves as the first qualitative exploration into diverse nicotine users’ perspectives about using a government-funded cessation site for quit support. Given that most tobacco cessation sites have been created to support adults (aged ≥19 years) with quitting cigarette use, it is essential to understand how to adapt these sites to effectively respond to the new tobacco use landscape, particularly with the increased emergence of individuals who vape and individuals who dual use. The results of this study provide important directions for updating cessation sites, so that they resonate with a variety of tobacco users, which will support the credibility and saliency of these sites, and ultimately improve the health and well-being of all nicotine users.

The findings of this study indicate that individuals want as much information as possible to support their cessation. However, what was critically important to participants was how a program delivers this information. Relying primarily on written information dissemination was perceived as out of date and nonengaging. Text-based information also appears to have the potential to inadvertently perpetuate inequities (eg, individuals with low literacy) according to a recent study [27]. Researchers examined the readability of 464 web pages that contained e-cigarette–related information, and they found that <25% of the web pages met the readability guidelines (at or below the sixth-grade reading level) regardless of target audience, message valence, or web page source [27]. In addition to the need for consideration in readability when providing this information, graphics, interactive features, engaging activities, and integration with social media and an app were described by participants as important steps moving forward. Furthermore, in this study, all nicotine users (36/36, 100%), but especially individuals who vape—who tended to be of a young age demographic—repeatedly described a need to rapidly view information and quickly access information and support via myriad ways (eg, live chat, telephone, graphics, and video clips), which speaks to the generational differences among nicotine users with respect to digital literacy—younger generations are accustomed to receiving fast and interactive information. Studies have shown that web-based content that is more interactive and engaging receives more attention and engagement than static content [28,29]. For example, Gao et al [29] found that antivaping posts on Instagram that were presented in a more engaging way, such as through memes, received more attention and engagement. This increase in attention and engagement has been shown to positively influence abstinence and cessation rates, which emphasizes the importance of paying attention to how content is delivered [28,30-33].

Our study findings also reveal that users’ desires for information have changed with the landscape of nicotine use; individuals who smoke, individuals who vape, and individuals who dual use unanimously demonstrated preference for the breadth and depth of information provided, but all participants (36/36, 100%)—individuals who vape in particular—described a desire for more vaping-specific information, including but not limited to biopsychological mechanisms and effects on the brain, harms, comparisons with combustible cigarettes, and vaping-specific quit strategies. In a recent systematic review of literature regarding the outcome of e-cigarette cessation, the authors found that there was very little information about e-cigarette cessation or tested strategies for e-cigarette cessation, and they concluded that there is an urgent need for addressing this gap [34]. This finding was validated in this study and was identified as a major gap among individuals who vape. As such, there may also be a missed opportunity to provide more comprehensive support for e-cigarette cessation on a trusted cessation website, such as QuitNow.

User-driven content appears to be a cornerstone of how individuals assess websites and programs, ultimately determining their choice to engage. Individuals who smoke, individuals who vape, and individuals who dual use described a desire to read stories from others, both about their quit journey and about their experiences and review of using web-based cessation supports, such as QuitNow. This aligns with the findings from Baumel and Kane [35], which showed a positive correlation between user reviews and increased and sustained use of self-guided eHealth interventions.

Participant accounts also highlight how creating a community of connection within a cessation resource must take into account the unique needs with regards to age. In particular, both individuals who vape and individuals who dual use in this study highlighted the need to make resources more youth led and youth inclusive, which aligns with trends in high e-cigarette uptake and use among the younger demographics. Similarly, several individuals who vape (4/12, 33%) in this study highlighted a dislike for the increased emphasis on including parents, largely owing to negative consequences, such as being shamed for vaping. This speaks to the importance of providing resources to allow for inclusion of a lateral and diverse support system outside the immediate family members and across settings. Similarly, individuals who dual use in this study uniquely discussed a desire for more self-directed support, such as tools for goal tracking, incentives, and resources to build their own self-awareness and sense of accountability while keeping stigma and shame to a minimum. Individuals who vape and individuals who dual use being particularly keen on receiving professional support, especially through chatting with a professional quit coach, may indicate that they have a stronger desire for quit support outside their immediate networks (eg, family and friends) for reasons outlined earlier.

Another noteworthy finding of this study was the contrast between extrinsic and intrinsic supports desired by different user groups. Although all types of users were interested in incentives to quit, individuals who vape were the only group to suggest a more intrinsic approach to quitting, specifically through mindfulness features. The reason behind this may be related to the fact that individuals who vape tend to be the youngest in age when compared with other nicotine users, with associated generational traits and historical factors possibly including increased autonomy owing to increased digitization of daily life experiences, fear of letting others down (Struik et al [36]), and increased conversation surrounding and use of mindfulness and similar intrinsic self-development tools over the past decade [37,38]. Alternatively, as mentioned by individuals who vape in this study, the prominent scarcity of targeted and engaging cessation supports and recruitment tools for e-cigarette use (vaping) may be leaving individuals who vape struggling to find appropriate extrinsic supports and thereby relying on intrinsic tools and strategies to cope and quit.

The findings also highlight the need for novel, digital methods for cessation support delivery. Most participants (21/36, 58%) described the necessity of having a smartphone app in conjunction with the QuitNow website. Users described myriad tools that may be of use through an app, including puff or cost trackers; games; reminders; notifications about tips, tricks, or fun facts; and opportunities to connect with other users. This finding is complemented by previous studies, whereby it has been found that using a smartphone app in partnership with standard cessation treatment yields high continuous abstinence rates, high individual retention rates, and improved nicotine withdrawal symptoms [31]. A smartphone app in conjunction with a website-delivered program is likely a feasible and useful option for enhancing current web-based smoking cessation programs.

### Comparison With Previous Studies

Ensuring that a cessation site was backed by a credible organization (eg, government) was important to users. This is consistent with previous qualitative research findings, whereby young adults reported trust in a smoking cessation app owing to its backing by credible institutions (eg, government and university) [36]. Participants reported that this backing lent to the belief that the app was genuinely built to help them and that it was based on credible evidence [36]. Our findings extend this evidence; the importance of credibility not only similarly applies across mobile and web-based platforms for smoking cessation but also for the cessation of a broad range of nicotine-related products and across diverse population groups. Furthermore, incorporating user reviews in the website was another endorsed suggestion to enhance credibility that was particularly shared among individuals who vape.

Social support was consistently a top reason for using the QuitNow site. Social support has been established as a critical component of any smoking cessation program [39]. Our findings align with the suggestions of previous studies calling for more attention to the social support features embedded in a program and the opportunities to leverage this component of a program [40]. For example, participants in this study indicated that a tagging system for the discussion content of the forum would be beneficial, so that they can receive more tailored support, which confirms the recommendations made previously [40].

Participants in this study wanted tailored information (eg, vaping cessation for youth should look different from vaping cessation support for adults). This is not new in the literature about tobacco control. According to review evidence (eg, the studies by Berg et al [41] and Graham et al [28]), interventions that take advantage of web-based technologies and the ability to tailor content to user demographics are critical to address the complexity that surrounds tobacco use currently. In this regard, smoking cessation websites, such as QuitNow, would do well to ensure that content is strategically organized and delivered to meet the needs of diverse end users, which no longer consists of primarily adult individuals who smoke.

Finally, contrary to previous literature, this study did not discover any significant differences between the cessation desires of men and women with respect to likes, dislikes, and suggestions surrounding QuitNow’s look, feel, content, and features. This may be owing to several reasons. First, the nature of the interview questions themselves (eg, focus on preferences and perspectives surrounding a specific support) may have diminished participant discussion surrounding their individual contextual experiences with respect to cessation outside their opinion about QuitNow. Sex and gender differences that are discussed in the published literature (eg, nicotine use and successful quit rates or differential pharmacological responses such as depression and weight loss) were not discussed in detail within participant accounts in this study and were not explicitly asked for within the study interview questions [13,14,16]. Second, the nature of the QuitNow website itself and the associated participant opinions may have masked the nuances; for example, although some studies suggest that women tend to present a greater need for social support than men, men also desire some level of social support [42]. Therefore, it is possible that the level of social support provided on QuitNow was below the desired threshold for the entire sample as all participants (36/36, 100%) across the sample highlighted the need for improving this feature. Furthermore, the historical context of the ongoing COVID-19 pandemic throughout the study time frame and the resultant increased isolation and reliance on digital interfaces may have further masked preexisting differences [43].

### Limitations and Strengths

This study has several strengths. First, the use of in-depth qualitative interviews with a diverse sample captured a variety of individual nuances within themes and enhanced the transferability of the findings to other populations. In addition, participants represented a diverse range of users with respect to age, nicotine user type, and indigeneity. Finally, most participants (32/36, 89%) were not QuitNow users, which decreased the chance of social desirability bias when describing the website. However, this could be viewed as a limitation to the study in that participants may have spent limited time in reviewing the QuitNow website and, thus, might be unaware of some features. Another limitation is the differing interfaces (web vs mobile) of the QuitNow website, which may have affected how users may have been able to view the site, thereby influencing how they may have felt about the site’s layout, look, feel, content, and so on. Similarly, many users (11/36, 31%) shared that they believed they had to create an account to navigate the website, which may have negatively affected the awareness about free supports available on the website. A key limitation of this study is the lack of diversity within the participant pool with respect to gender: no nonbinary or gender-nonconforming individuals self-selected within the large participant pool, which may speak to a lack of inclusive targeting tools used in the recruitment materials. This, combined with the surprising lack of gender-specific differences between men and women within the findings, suggests the need for including a diverse array of gender-inclusive factors in cessation studies and supports, such as in building inclusive recruitment materials, using components that comprehensively inquire for gender-related influences in interviews, and comprehensively translating gender-specific findings into supports [44]. In addition, this study’s sample size and selection strategy may also have masked the qualitative differences between genders that may have existed. In addition, we did not compare gender differences within nicotine user subgroups. As such, future qualitative studies with a large participant pool and quantitative or mixed methods studies that do this may provide great insight into gender differences in cessation support needs across, between, and within a diverse array of nicotine users. A final limitation is that the study population was from British Columbia, Canada, only. As such, the transferability of the findings to non-BC and non-Canadian contexts may be limited.

### Conclusions

In this study, we provide directions for enhancing the saliency of web-based cessation programs for a variety of tobacco use behaviors that hallmark current tobacco use. We have outlined several steps for leveraging the strengths of existing cessation programs and for addressing current gaps. These adaptations will not only enhance the reach of web-based cessation programs, such as QuitNow, but will also ensure its relevance to the shifting behaviors and population needs as it relates to tobacco use.

## Data Availability

The data sets generated and analyzed during this study are available from the corresponding author upon reasonable request.
